# Anandamide and WIN 55212–2 Afford Protection in Rat Brain Mitochondria in a Toxic Model Induced by 3-Nitropropionic Acid: an In Vitro Study

**DOI:** 10.1007/s12035-024-03967-2

**Published:** 2024-02-03

**Authors:** Marisol Maya-López, Luis Angel Monsalvo-Maraver, Ana Laura Delgado-Arzate, Carolina I. Olivera-Pérez, Mohammed El-Hafidi, Alejandro Silva-Palacios, Omar Medina-Campos, José Pedraza-Chaverri, Michael Aschner, Alexey A. Tinkov, Isaac Túnez, Socorro Retana-Márquez, Cecilia Zazueta, Abel Santamaría

**Affiliations:** 1 Doctorado en Ciencias Biológicas y de La Salud, Universidad Autónoma Metropolitana, 09310 Mexico City, Mexico; 2 Facultad de Ciencias, Universidad Nacional Autónoma de México, 04510 Mexico City, Mexico; 3 Departamento de Biomedicina Cardiovascular, Instituto Nacional de Cardiología, SSA, 14080 Mexico City, Mexico; 4 Laboratorio F-315, Departamento de Biología, Facultad de Química, Universidad Autónoma de México, 04510 Mexico City, Mexico; 5 Department of Molecular Pharmacology, Albert Einstein College of Medicine, Bronx, NY 10461 USA; 6 Laboratory of Molecular Dietetics, IM Sechenov First Moscow State Medical University (Sechenov University), Moscow, 119435 Russia; 7 Department of Human Ecology and Bioelementology, and Department of Medical Elementology, Peoples’ Friendship University of Russia (RUDN University), Moscow, 117198 Russia; 8 Instituto de Investigaciones Biomedicas Maimónides de Córdoba (IMIBIC), Córdoba, Spain; 9 Departamento de Bioquímica y Biología Molecular, Facultad de Medicina y Enfermería, Universidad de Córdoba, Córdoba, Spain; 10 Red Española de Excelencia en Estimulación Cerebral (REDESTIM), 14071 Córdoba, Spain; 11 Departamento de Biología de La Reproducción, Universidad Autónoma Metropolitana-Iztapalapa, 09310 Mexico City, Mexico

**Keywords:** Brain mitochondria, Mitochondrial dysfunction, Succinate dehydrogenase inhibition, Mitochondrial cannabinoid receptor 1 regulation, Endocannabinoid, Protective regulatory activity

## Abstract

Mitochondrial dysfunction plays a key role in the development of neurodegenerative disorders. In contrast, the regulation of the endocannabinoid system has been shown to promote neuroprotection in different neurotoxic paradigms. The existence of an active form of the cannabinoid receptor 1 (CB1R) in mitochondrial membranes (mitCB1R), which might exert its effects through the same signaling mechanisms as the cell membrane CB1R, has been shown to regulate mitochondrial activity. Although there is evidence suggesting that some cannabinoids may induce protective effects on isolated mitochondria, substantial evidence on the role of cannabinoids in mitochondria remains to be explored. In this work, we developed a toxic model of mitochondrial dysfunction induced by exposure of brain mitochondria to the succinate dehydrogenase inhibitor 3-nitropropionic acid (3-NP). Mitochondria were also pre-incubated with the endogenous agonist anandamide (AEA) and the synthetic CB1R agonist WIN 55212–2 to evaluate their protective effects. Mitochondrial reduction capacity, reactive oxygen species (ROS) formation, and mitochondrial swelling were assessed as toxic markers. While 3-NP decreased the mitochondrial reduction capacity and augmented mitochondrial ROS formation and swelling, both AEA and WIN 55212–2 ameliorated these toxic effects. To explore the possible involvement of mitCB1R activation on the protective effects of AEA and WIN 55212–2, mitochondria were also pre-incubated in the presence of the selective CB1R antagonist AM281, which completely reverted the protective effects of the cannabinoids to levels similar to those evoked by 3-NP. These results show partial protective effects of cannabinoids, suggesting that mitCB1R activation may be involved in the recovery of compromised mitochondrial activity, related to reduction of ROS formation and further prevention of mitochondrial swelling.

**Supplementary Information:**

The online version contains supplementary material available at 10.1007/s12035-024-03967-2.

## Introduction

Mitochondria are the main suppliers of neural energy and play a critical role in the brain in the metabolism of neurotransmitters [1], as well as in axonal communication, survival, and growth [2]. Mitochondria also participate in cellular bioenergetics, providing such energy through oxidation–reduction processes carried out by the electron transport chain (ETC), that results in O_2_ reduction and in the establishment of a proton electrochemical gradient across the inner mitochondrial membrane which impulse adenosine triphosphate (ATP) synthesis [3]. Mitochondrial quality control is maintained by dynamic processes which include biogenesis, fission/fusion, and mitophagy [4]. When phosphorylation decreases, electrons’ leaking from the ETC partially reduces oxygen to superoxide radical (O_2_^·−^), which can be spontaneously or enzymatically converted to H_2_O_2_ under normal conditions, acting as a second messenger [5]. However, when reactive oxygen species (ROS) levels are high, they lead to oxidative stress and eventually cell death in a variety of pathological conditions, including hypoxia [6], ischemia and reperfusion [7], aging, and neurodegeneration [8, 9].

Although cells and mitochondria possess antioxidant systems that neutralize ROS production [10], when the rate of ROS formation overwhelms the antioxidant defense, mitochondria become the first target of their action, resulting in swelling, excessive ROS formation, disruption of calcium (Ca^2+^) homeostasis, loss of reduction capacity, decreased electron transport activity, reduced ATP synthesis, compromised mitochondrial dynamics, and enhancement of pro-apoptotic factors release [11, 12]. These events trigger major mechanisms of cell death, thus leading to the development of neurodegenerative diseases [13, 14].

3-Nitropropionic acid (3-NP) is a mycotoxin employed in biomedicine to produce a toxic model by inducing neural cell death that recapitulates several features of HD such as mitochondrial dysfunction, leading to GABAergic cell degeneration in the striatum [15]. This toxic compound is an isoelectric analog of succinate, a Krebs’ cycle metabolite and substrate for succinate dehydrogenase (SDH, mitochondrial Complex II). 3-NP irreversibly inhibits SDH activity, thus decreasing the ETC activity, ATP synthesis, membrane mitochondrial potential (Δψm), and oxygen consumption, while increasing ROS formation, mitochondrial permeability transition pore (mPTP) opening, and mitochondrial swelling [16–18].

The endocannabinoid system (ECS) is responsible for the coordination of several physiological events in living organisms, including the inhibition of chronic pain and regulation of appetite and sleep cycle, to name a few [19]. In the central nervous system (CNS), the ECS participates in the regulation of neurotransmission, memory, cognition, and locomotor activity [20]. This system is composed of endogenous ligands, canonic receptors (cannabinoid receptors 1 and 2, or CB1R and CB2R, respectively) and other receptors (G55 protein coupled receptor or GPR55, and vanilloid transient receptor potential V1 or TRPV1), and synthesis and degradative enzymes for endocannabinoids. CB1R and CB2R are widely expressed in neurons and glial cells (microglia and astrocytes), specifically in the cortex, hippocampus, cerebellum, midbrain, and basal ganglia [21]. Endogenous ligands for these receptors are fatty acid amides, such as N-arachidonoylethanolamine (anandamide or AEA) and 2-arachidonoyl glycerol (2-AG). AEA has been shown to exert effects like those reported for phytocannabinoids found in *Cannabis* sp., and is responsible for the regulation of appetite and mood [22].

CB1R is located in synapses, both on the presynaptic and postsynaptic membranes, where it modulates the release of neurotransmitters and the activity of postsynaptic receptors [23, 24]. These receptors are G-coupled proteins typically acting through the inhibition of adenylate cyclase, decreasing Ca^2+^ currents and cyclic adenosine monophosphate (cAMP), and increasing K^+^ currents [25]. Through these actions, CB1R regulates cognitive and locomotor activity in the cortex, hippocampus, and basal ganglia [26]. CB1R is also found in intracellular compartments such as endosomes and outer mitochondrial membranes (mitCB1R) [27, 28]. Precisely, mitCB1R modulates neural functions related to energy metabolism, axonal transport, and synaptic transmission through the inhibition of adenylate cyclase and protein kinase 1, finally regulating the activity of the ETC [29]. The assumed physiological nature of these events is related to the inhibition of excitatory transmission, the preservation of Ca^2+^ homeostasis, and the modulation of ATP production for the regulation of memory in the hippocampus [30]. The preferential reduction in the activity of mitochondrial Complex I (NADH: ubiquinone oxidoreductase) by mitCB1R is related to the modulation of oxidative phosphorylation in hippocampal neurons [31], which in turn reduces hippocampal synaptic transmission to regulate partial memory, as demonstrated in mice receiving the synthetic CB1R agonist WIN 55212–2 [32]. However, the concept that mitCB1R downregulates mitochondrial activity, which is crucial for the preservation of vital functions of neural cells, might suggest in the first place, the potential risk of redox dysfunction, Ca^2+^ influx deregulation, and critical ATP deficit [33]. Thus, an important question remains as to the precise role of these receptors under pathological conditions. This is particularly relevant considering that activation of CB1R induces neuroprotective effects in in vitro and in vivo toxic paradigms [34, 35]. In this regard, it is noteworthy that AEA (50 µM), a CB1R endogenous agonist, has been shown to increase mitochondrial swelling while it decreases mitochondrial functionality and membrane potential per se in mice liver mitochondria in a mechanism apparently independent of mitCBR1, though it exhibited protective properties by ameliorating the Ca^2+^-induced swelling and cytochrome release [36], thus contributing to the controversy on the role of the ECS in mitochondrial activity. In addition, it has been demonstrated that the synthetic cannabinoid receptor agonists CP55940 and JWH-015 can protect rat cortical mitochondria from paraquat-induced oxidative stress and mitochondrial dysfunction, also reducing the Ca^2+^-induced swelling apparently through radical-scavenging properties, thus endorsing protective effects of cannabinoids in toxic paradigms, though no approaches to a possible involvement of mitCB1R activation in these effects have been assessed to date [37]. Moreover, biphasic activity of mitochondrial complexes has been reported when several CB1R natural (endocannabinoids and phytocannabinoids) and synthetic agonists and antagonists were tested in pig brain mitochondria [38], suggesting that different agents might evoke differential responses in the regulation of these receptors. Combined, this evidence sustains the need of additional studies aimed to characterize the precise role of cannabinoids and mitCB1R activation in physiological and pathological conditions in brain mitochondria. Therefore, in this study, we exposed isolated mitochondrial obtained from the rat brain to 3-NP and challenged this condition with the endocannabinoid AEA, the synthetic cannabinoid WIN 55 212–2, and/or the CB1R antagonist AM281 to explore the possible involvement of mitCB1R activation in a toxic model of mitochondrial dysfunction induced by SDH inhibition. Three toxic endpoints were assessed: loss of mitochondrial reduction capacity, ROS formation, and mitochondrial swelling. Our results demonstrate that endogenous and synthetic cannabinoids exert protective effects in the toxic model studied, suggesting that activation of mitCB1R is involved in these effects.

## Materials and Methods

### Animals

Adult male Wistar rats (250–300 g; *N* = 40) were obtained from the vivarium of the Instituto Nacional de Cardiología *Ignacio Chávez* and the Universidad Autónoma Metropolitana-Iztapalapa. All animals had access to food and water ad libitum, and were maintained under controlled conditions of light (12:12 h of light/darkness), temperature (25 °C), and humidity (50%) until they were used for the experiments. All experimental procedures were carried out following the guidelines of NOM-062 and local committees on the ethical use of animals. The project was evaluated and officially registered under the protocol 73/20 (INNN).

### Reagents

3-Nitropropionic acid, arachidonylethanolamide (AEA or anandamide), 1-(4,5-dimethylthiazol-2-yl)-3,5-diphenyl formazan (MTT), WIN 55212–2, succinic acid, antimycin, calcium chloride (CaCl_2_), sucrose, HEPES, dichlorofluorescein (DCF), EGTA, horseradish peroxidase, and H_2_O_2_ were all obtained from Sigma-Aldrich (Sigma Chemical Co.; Saint-Louis, MO). AM281 and rotenone were obtained from Tocris Bioscience (Bristol, UK), and Bovine Serum Albumin Fraction V Fatty Acid-Free Fisher was obtained from MP Biomedicals (Fisher Scientific; Hampton, NH). All other reagents were obtained from known commercial sources and were of analytical grade. Except for AEA (prepared in EtOH) and AM281 (prepared in DMSO), all reagents were prepared with distilled water.

### Isolation of Rat Brain Mitochondria

The mitochondrial isolation procedure was carried out following a previous protocol [39], with some modifications. Rats (*n* = 3–12 per experimental protocol) were euthanized by decapitation and their brains were dissected out and homogenized in mitochondrial isolation buffer (MIB; 250 mM sucrose, 10 mM HEPES, 1 mM EGTA dissolved in 1–5 M KOH, pH 7.2). The homogenized samples were centrifuged at 3500 rpm for 10 min at 4 °C, using a Sorvall Superspeed RC2-B ultracentrifuge. The obtained supernatants were filtered and centrifuged at 10,000 rpm for 10 min at 4 °C; the pellets were resuspended in 1 mL of 0.3% albumin for 10 min and MIB was added; next, samples were centrifuged at 10,000 rpm for 10 min at 4 °C. The resulting mitochondrial pellets were resuspended in 200 μL of MIB and recollected to assess volume. The protein concentration in the samples was quantified by Biuret’s method. In addition, to assess the functionality of the isolated mitochondria, a pilot study measuring oxygen consumption was evaluated as an index of mitochondrial coupling, according to a method previously described [40]. Results of this assay are shown as supplementary materials to this article.

### Assessment of Mitochondrial Reduction Capacity Through Succinate Dehydrogenase Activity

To evaluate the mitochondrial reduction capacity from energized Complex II, we used succinate as a substrate along with rotenone (a Complex I inhibitor). Rotenone prevents reverse electron transfer through this complex and blocks oxaloacetate accumulation, which in turn inhibits Complex II activity. Mitochondrial pellets (150 μg) were used fresh and incubated in 200 μL MIB for 60 min; in such a time period, experimental treatments were added gradually, as summarized in Table 1. Vehicles for AEA and AM281 (EtOH and DMSO) were tested during the experimental setup. None of these solvents produced any effect on the basal MTT reduction capacity at the conditions employed (data not shown).

**Table 1:**
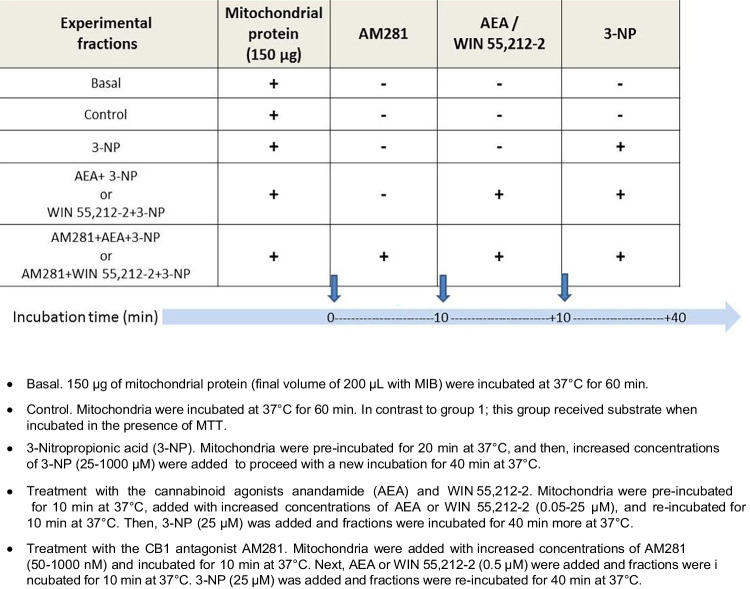
Experimental conditions in mitochondrial fractions

Mitochondrial reduction capacity was assessed according to a previous report [41]. Briefly, following the incubation of mitochondrial fractions with the different experimental conditions, the substrate for SDH, succinate (10 mM), and the Complex I inhibitor, rotenone (1 μM) was added to the medium together with MTT (1.2 mM; used from a previously prepared stock containing 5 mg in 1 mL MIB), and fractions were then re-incubated for 20 min at 37 °C. At the end of incubation, samples were centrifuged at 12,000 rpm for 3 min at 4 °C, and then pellets were resuspended in 200 μL acid-alcohol solution (27 mL propanol plus 3 mL X-100 Triton plus 25 µL HCl concentrated). Optical density of all samples was assessed at 570 nm in a transparent 96-well plate in a Biotek Synergy—HTX multimodal plate reader (Agilent, CA, USA) equipment.

### Assessment of H_2_O_2_ Formation by Fluorescence

Detection of H_2_O_2_ was assessed as an index of ROS formation, according to a method previously described [42]. In total, 100 μg of protein from the mitochondrial pellets was collected and incubated with 250 μL buffer for ROS detection (100 mM sucrose, 75 mM KCl, 5 mM Tris Base, 3 mM MgCl_2_, 1 mM KH_2_PO_4_, and 10 μM EGTA, at pH 7.4) for 60 min (a time period in which different compounds were gradually added to define the experimental conditions already described in Table 1) and incubated at 30 °C. At the end of incubation, samples (250 μL total volume) were deposited in a dark 96-well plate and the fluorescence signals in all fractions were read at *λ*_ex_ = 475 nm ± 4 nm and *λ*_em_ = 525 nm ± 4 nm in a Biotek Synergy HXT multimodel plate reader (Agilent, CA, USA) in the presence of 1 μg horseradish peroxidase plus 1 μM DCF. Initial fluorescence detection was carried out for 1 min, and then, 10 mM succinate was added and samples were read for 7 min more. Then, 5 μM antimycin was added to samples and the reading was followed for 7 min more. Experimental conditions assessed in this assay are summarized in Table 2.

**Table 2:**
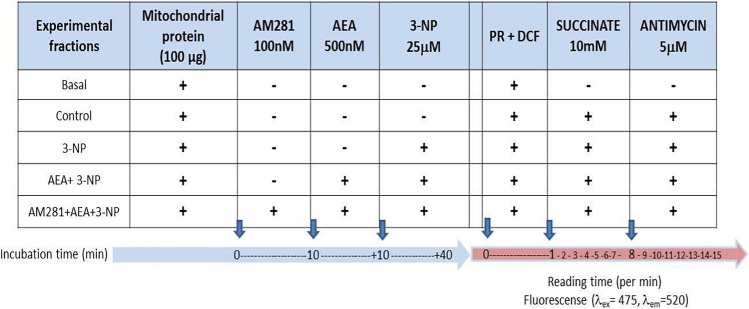
Experimental conditions tested in mitochondrial fractions to assess ROS formation

### Mitochondrial Swelling Assay

Mitochondrial swelling was assessed according to a previous protocol [43], as an index of the opening mitochondrial permeability transition pore (mPTP) in the presence of a Ca^2+^ overload and 3-NP as the inducing agent. The decrease in absorbance at 540 nm reflects the increase in the matrix volume. Once obtained, the mitochondrial protein pellets isolated from the rat brain (250 µg protein) were completed to a final volume of 200 µL with mitochondrial swelling buffer (MSB) containing 200 mM sucrose, 10 mM MOPS, 0.05 mM EGTA, and 10 mM KH_2_PO_4_, pH 7.4 plus 10 mM succinate as substrate, 1 μM rotenone for Complex I inhibition, and 25 μM 3-NP. Some mitochondrial fractions were pre-treated with 500 nM AEA and/or 100 nM AM281, and optical density was measured at 540 nm following the addition of increased concentrations of CaCl_2_ (starting from 0 to gradually reaching 80, 120, and finally 160 μM), reading the optical density for 5 min every minute before increasing the Ca^2+^ concentration. Normalized values of absorbance were used to determine the percentage of swelling compared to initial absorbance in non-exposed Ca^2+^ mitochondria.

### Statistical Analysis

All results were normalized to the baseline mean value and analyzed using a one-way analysis of variance (ANOVA), followed by post hoc Tukey’s test for multiple comparisons among treatments. In the case of the ROS assay, mean values of slopes obtained from a H_2_O_2_ curve were compared, and lineal regression was employed. For all experiments,* n* = 3–12 experiments per group were considered. Values of *p* ≤ 0.05 were considered as statistically significant.

## Results

### 3-NP Decreased Mitochondrial Activity in a Concentration-Dependent Manner, While CB1R Agonists Did Not Affect This Endpoint

In isolated mitochondrial, MTT reduction has been associated with the mitochondrial reduction capacity mediated by the activity of dehydrogenases in the respiratory chain. To evaluate the effect of 3-NP (Complex II inhibitor) on mitochondrial reduction capacity, succinate was used as an electron donor, whereas rotenone served to inhibit the return transfer of electrons from Complex II to Complex I, thus ensuring direct migration of electrons to Complexes III and IV.

**Fig. 1:**
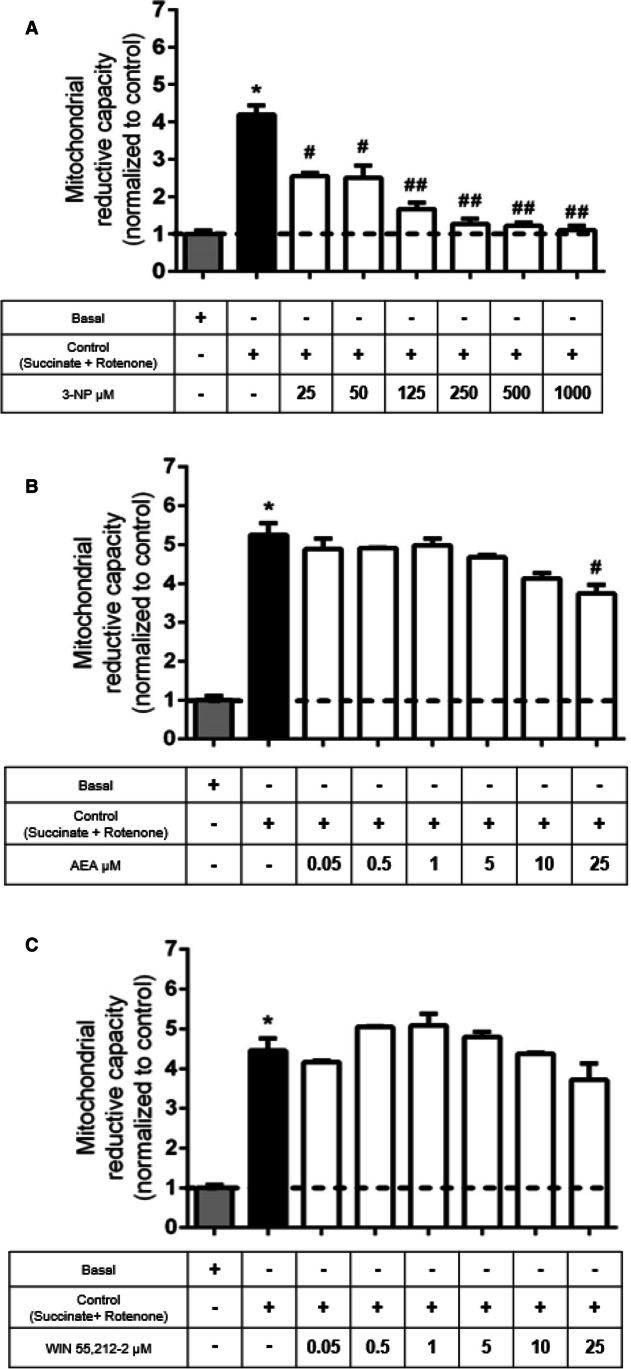
In **A**, the concentration–response effect of 3-nitropropionic acid (3-NP) on mitochondrial reduction capacity in rat brain mitochondria. Bars represent mean values ± SEM of *n* = 3 experiments per group. *p ≤ 0.0001, different from the basal condition (mitochondria incubated only with MIB; gray bar); ^**#**^*p* ≤ 0.001 and ^**##**^*p* ≤ 0.0001, different from control mitochondria (black bar). One-way ANOVA followed by Tukey’s test. In **B** and** C**, the concentration effects of anandamide (AEA) and WIN 55212–2, respectively, on mitochondrial activity. Bars represent mean values ± SEM of *n* = 3 experiments per group. *p ≤ 0.0001, different from the basal condition (mitochondria incubated only with MIB; gray bar); ^**#**^*p* < 0.05 different from control. One-way ANOVA followed by Tukey’s test

Figure 1A shows the concentration effect of 3-NP on the decrease in mitochondrial reduction capacity. MTT reduction mediated by succinate oxidation decreased with all 3-NP concentrations (25–1000 µM, with 40% decrease at 25 µM; *p* ≤ 0.001). The half maximal effective concentration (EC_50_) was near 25 μM (representing 40% of loss of mitochondrial function).

In a second experiment, mitochondria were treated with increased concentrations of AEA or WIN 55212–2 (0.05, 0.5, 1, 5, 10, and 25 μM) to explore the effects of these agents on mitochondrial activity. In a 50–5000-nM concentration range, neither AEA nor WIN 55212–2 modified the mitochondrial reduction capacity, compared with control mitochondria (Fig. 1B and C, respectively). AEA decreased this function only at 25 μM concentration, while WIN 55212–2 did not affect this endpoint. These results demonstrate that these cannabinoids have innocuous effects on the mitochondrial reduction capacity when used at physiological (nM) concentrations, whereas at supraphysiological concentrations, they exert adverse effects. Based on these results, we demonstrated that cannabinoids are safe to test in our mitochondrial function experiments at the evaluated concentrations. All results appear normalized with respect to the basal condition.

### Pre-treatment of Mitochondria with AEA or WIN 55212–2 Attenuated the Decrease in Mitochondrial Reduction Capacity Induced by 3-NP

**Fig. 2:**
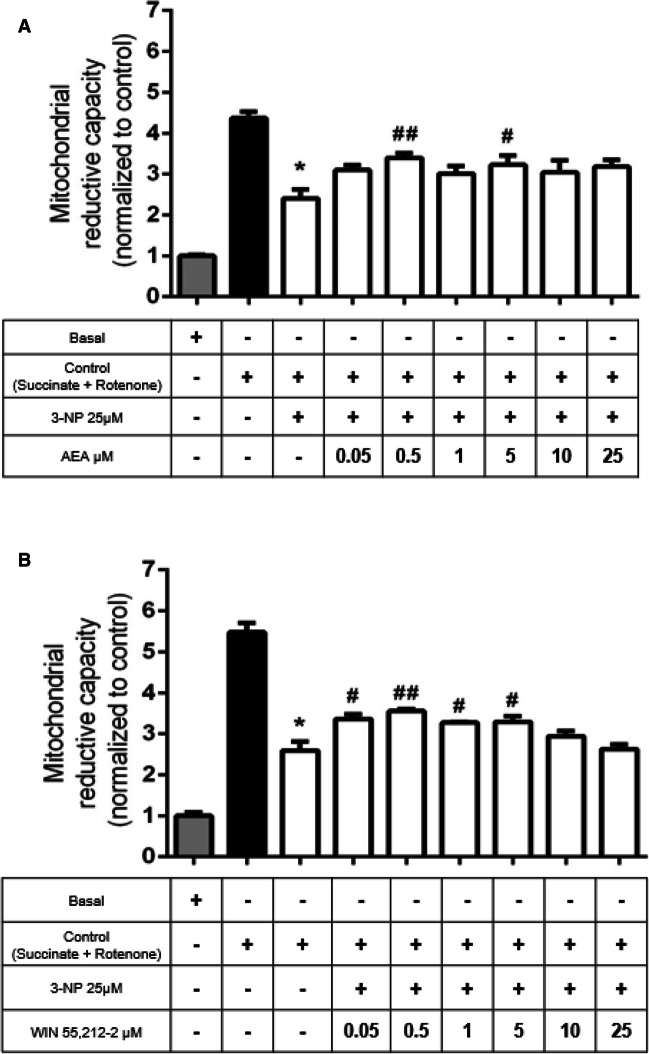
Concentration–response effects of anandamide (AEA; in **A**) and WIN 55212–2 (in **B**) on the 3-nitropropionic acid (3-NP)-induced decrease in mitochondrial reduction capacity in rat brain mitochondria. Bars represent mean values ± SEM of *n* = 5–8 experiments per group. *p ≤ 0.0001, 3-NP different of control mitochondria (black bar); ^**#**^*p* ≤ 0.05 and ^**##**^*p* ≤ 0.01, different from 3-NP (white bars). One-way ANOVA followed by Tukey’s test

We further evaluated the preventive effect of increased concentrations of AEA (Fig. 2A) or WIN 55212–2 (Fig. 2B) on the mitochondrial reduction capacity of mitochondria exposed to 25 μM 3-NP to find the optimal protective concentrations of these agents. AEA recovered partially the levels of MTT reduction significantly at 0.5 and 5 μM (41% (*p* ≤ 0.01) and 34% (*p* ≤ 0.05), respectively), compared to 3-NP-treated mitochondria (Fig. 2A). In turn, WIN 55212–2 exhibited similar effects to those produced by AEA, showing partial prevention of loss of mitochondrial activity induced by 3-NP at concentrations of 0.05, 0.5, 1, and 5 μM (30% (*p* ≤ 0.05), 37% (*p* ≤ 0.01), 26% (*p* ≤ 0.05), and 27% (*p* ≤ 0.05), respectively) (Fig. 2B). Based on these results, we demonstrated that cannabinoids ameliorate the toxic effect of 3-NP in a partial manner. All results appear normalized with respect to the basal condition.

### The Partial Recovery of Mitochondrial Activity Induced by CB1R Agonists in 3-NP-Exposed Mitochondria Is Mediated by mitCB1R Activation

**Fig. 3:**
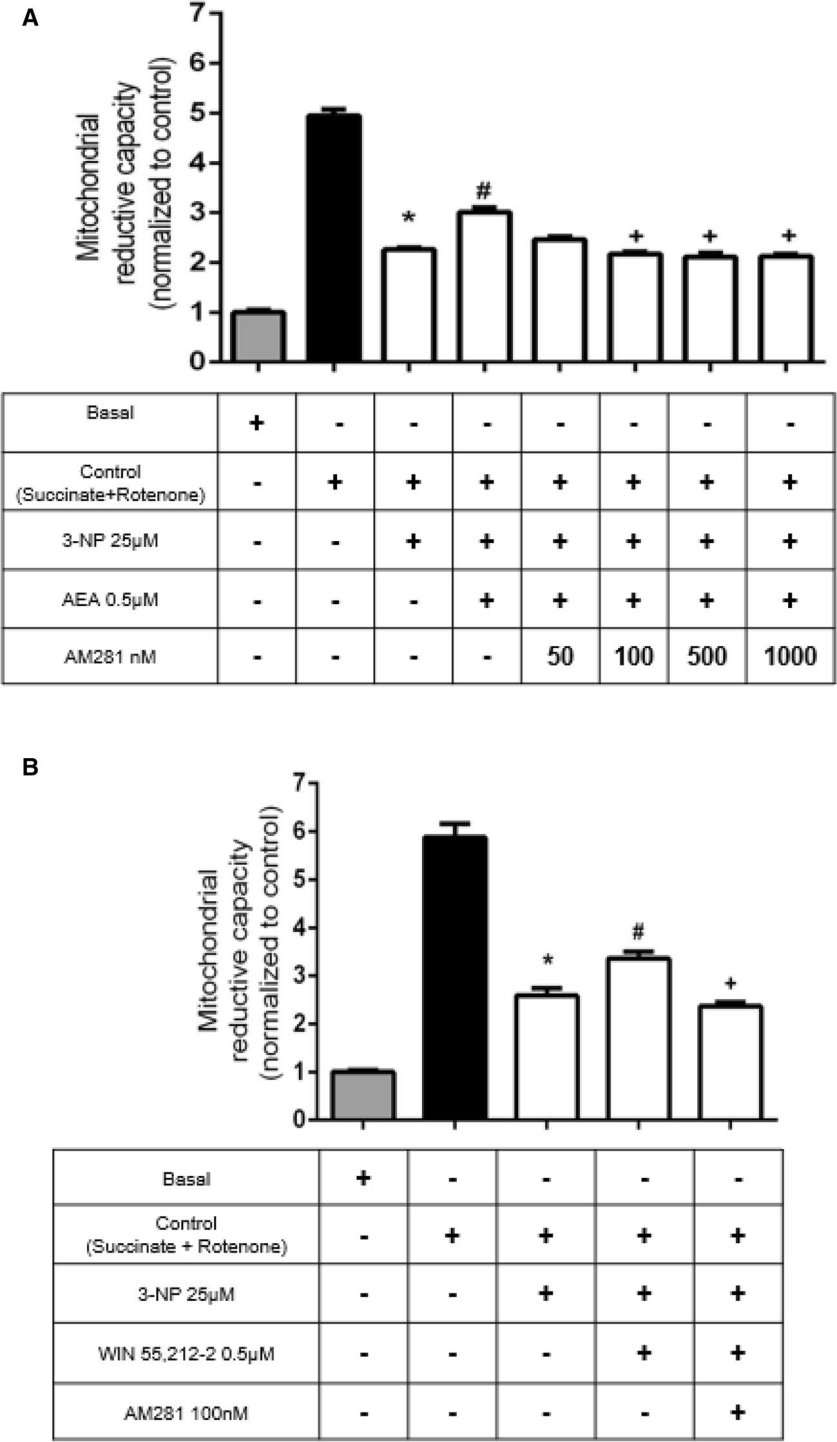
Effects of the selective CB1R antagonist AM281 on the protective efficiency of anandamide (AEA; in **A**) and WIN 55212–2 (in **B**) in the mitochondrial reduction capacity of 3-nitropropionic acid (3-NP)-exposed mitochondria. Bars represent mean values ± SEM of *n* = 5–8 experiments per group in **A**, and *n* = 4–5 in **B**. *p ≤ 0.0001, 3-NP different of control mitochondria (black bar); ^**#**^*p* ≤ 0.01, different from 3-NP (white bars); ^**+**^*p* ≤ 0.01, different from AEA + 3-NP and WIN 55212–2 + 3-NP. One-way ANOVA followed by Tukey’s test

To investigate the involvement of mitCB1R in the protective effects of AEA and WIN 55 212–2 in 3-NP-exposed mitochondria, we evaluated the effect of the CB1R receptor antagonist AM281 on mitochondrial reduction capacity (Fig. 3). The recovery of mitochondrial function induced by AEA in 3-NP-treated mitochondria decreased in the presence of AM281 at 100-, 500-, and 1000-nM concentrations (*p* ≤ 0.0001, different of AEA + 3-NP), reaching levels of MTT reduction similar to those produced by 3-NP alone (Fig. 3A). AM281 also prevented the effect of WIN 55212–2 on 3-NP-decreased MTT reduction at 100 nM (*p* ≤ 0.05), reaching levels of MTT reduction similar to those of 3-NP alone (Fig. 3B). At the concentration tested, AM281 per se had no effect on the basal mitochondrial reduction capacity (data not shown). These results demonstrate the involvement of mitCB1R in the regulation of the protective responses in mitochondria. All results appear normalized with respect to the basal condition.

### Complex I Participates in Mitochondrial Reduction Capacity Following mitCB1R Activation in Mitochondria Exposed to 3-NP

**Fig. 4:**
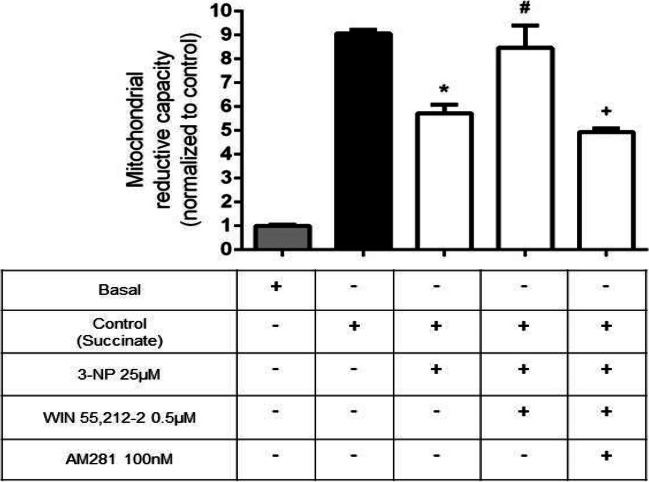
Effect of WIN 55212–2 on dehydrogenase activity (reduction capacity) of mitochondrial electron chain (experiment without rotenone). Bars represent mean values ± SEM of *n* = 5 experiments per group. *p ≤ 0.0001, different of control mitochondria; ^**#**^*p* ≤ 0.01, different from the 3-NP group; ^**+**^*p* ≤ 0.001, different from WIN 55212–2 + 3-NP. One-way ANOVA followed by Tukey’s test

To determine the contribution of all mitochondrial complexes in mitochondrial reduction capacity, additional experiments were carried out in the absence of rotenone. Figure 4 depicts a fourfold increase in baseline (control) MTT reduction when Complex I is active. 3-NP decreased this marker by 36% compared to the control value (*p* ≤ 0.0001), whereas the pre-treatment of mitochondria with 0.5 µM WIN 55212–2 recovered the reduction capacity up to levels similar to those of the control value (*p* ≤ 0.01 compared to 3-NP). This effect was related to mitCB1R activation, as pre-incubation of mitochondria with AM281 completely inhibited the protective efficiency of WIN 55212–2 (*p* ≤ 0.001). Again, AM281 per se had no effect on the basal mitochondrial reduction capacity (data not shown).

### Activation of mitCB1R Prevented 3-NP-Induced Formation of H_2_O_2_ in Mitochondria

**Fig. 5:**
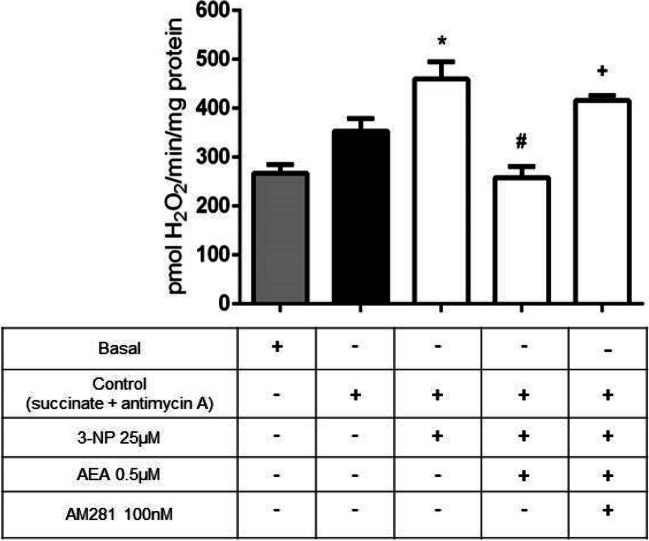
Effect of mitCB1R activation by AEA on H_2_O_2_ formation in rat brain mitochondria. The values of fluorescence obtained were compared against a standard curve generated by addition of known concentrations of H_2_O_2_ to the reaction buffer containing DCF, horseradish peroxidase, and mitochondria. Bars represent mean values ± SEM of *n* = 6–12 independent experiments. *p ≤ 0.05, different from control; ^#^*p* ≤ 0.0001, different from 3-NP; ^+^*p* ≤ 0.01, different from AEA + 3-NP. One-way ANOVA followed by Tukey’s test

Next, we determined the rate of H_2_O_2_ production as an index of ROS formation by measuring the fluorescence signal emitted by DCF in mitochondria. Figure 5 shows an increase in the levels of H_2_O_2_ induced by 3-NP (459 *vs.* 353 pmol H_2_O_2_/min/mg; *p* ≤ 0.05) through a mechanism involving succinate oxidation in the presence of antimycin. In turn, AEA decreased the 3-NP-induced H_2_O_2_ (258 pmol H_2_O_2_/min/mg; *p* ≤ 0.0001, different of 3-NP), whereas mitochondria preincubated with AM281 inhibited the protective effect of AEA (416 pmol H_2_O_2_/min/mg; *p* ≤ 0.01, different of AEA + 3-NP). These results suggest that the increase of H_2_O_2_ in the presence of the Complex III inhibitor plus 3-NP might have originated in a site located between the ubiquinone pool and the site of 3-NP binding in Complex II.

### AEA Prevents Mitochondrial Swelling Induced by 3-NP

**Fig. 6:**
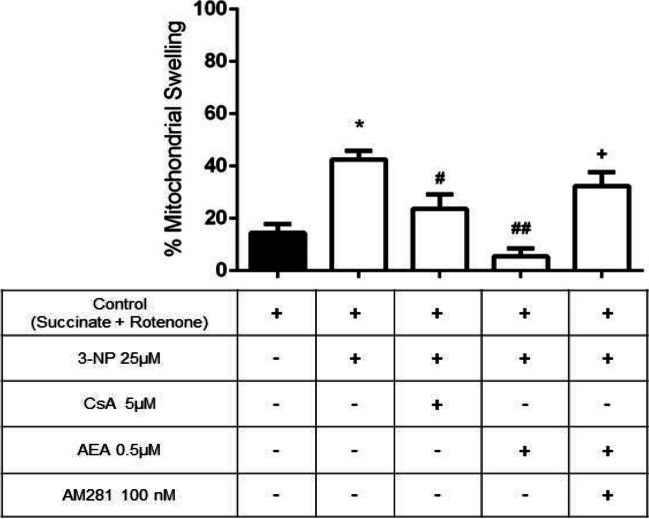
Effect of AEA on mitochondrial swelling induced by 3-NP. Bars represent mean values ± SEM of *n* = 7 independent experiments. *p ≤ 0.001, different of the control; ^#^*p* ≤ 0.05 and ^##^*p* ≤ 0.0001, different from 3-NP; ^+^*p* ≤ 0.001, different from AEA + 3-NP. One-way ANOVA followed by Tukey’s test

Mitochondrial swelling is an adverse event responsible for triggering the mPTP opening; this can be induced by Ca^2+^ overload and in the presence of 3-NP as an inducing agent. Mitochondria exposed to 25-µM 3-NP showed an increase in swelling of 40% (*p* ≤ 0.001) compared to control mitochondria (basal value of swelling of 14% following Ca^2+^ addition; Fig. 6), while AEA (500 nM) prevented the 3-NP–induced swelling in a significant manner (*p* ≤ 0.0001, compared to 3-NP). This protective effect was more prominent than that of 5-μM cyclosporin A (CsA), a well-known mPTP opening blocker (*p* ≤ 0.05, compared to 3-NP). In turn, AM281 effectively reverted the effect of AEA by increasing swelling to values similar to those of 3-NP (*p* ≤ 0.001, compared to AEA + 3-NP).

## Discussion

In this work, we evaluated the protective effects elicited by cannabinoid agents and the possible involvement of mitCB1R in a toxic model induced by 3-NP in isolated rat brain mitochondria. For this purpose, we used the endocannabinoid AEA and the synthetic cannabinoid WIN 55212–2, both well-known CB1R agonists. We also used AM281 as a pharmacological tool to inhibit the activity of mitCB1R. The mitochondrial reduction capacity, ROS formation, and mitochondrial swelling were all quantified to assess the protective capacity of these compounds.

To assess mitochondrial reduction capacity at the level of Complex II, succinate was used as substrate/electron donor for SDH, whereas rotenone was used as a well-known Complex I inhibitor to induce succinate oxidation. Reduction capacity was significantly decreased by 3-NP, while AEA and WIN 55212–2 partially prevented this effect, and this protection was likely to be mediated by mitCB1R, as evidenced by the effect of AM281. Noteworthy, in additional experiments in which mitochondria were incubated in the absence of rotenone, the cannabinoid-induced protective effect was complete, suggesting that, despite the toxic model employed specifically affects Complex II, an active role of Complex I is likely essential for the preservation and integral recovery of mitochondrial activity in 3-NP-exposed mitochondria.

In turn, AEA prevented the 3-NP-induced H_2_O_2_ formation in mitochondria through a protective mechanism likely to be dependent on mitCB1R activation, suggesting that, in this experimental model, the increased ROS formation induced by 3-NP might be inhibited by mitCB1R activation probably through the regulation of H_2_O_2_ formation from the mitochondrial respiratory chain; therefore, it is likely that mitCB1R participates in the modulation of ROS formation. In addition, our results show that mitochondrial swelling induced by 3-NP was prevented by AEA probably through the activation of mitCB1R, suggesting that this receptor may modulate the activity of mPTP via the regulation of Ca^2+^ channels.

Regarding the toxic model used in this study, 3-NP is a mycotoxin derived from the fungus species *Aspergillus* sp. and *Artrinium* sp., and plants such as *Astragalus* sp. and *Indigofera endecaphylla*. This toxin inhibits selectively the mitochondrial Complex II and has been employed to reproduce the pathological features of HD in animal models in vivo, as it triggers mechanisms of neural damage such as mitochondrial dysfunction, excitotoxicity, oxidative stress, inflammation, and neural cell death in the striatum, brain cortex, globus pallidus, and hippocampus, thus generating motor alterations in rodents [44, 45]. Altered striatal cell morphology has been observed in adult male rats lesioned with 3-NP, generating pyknosis, swelling, and striosomal alterations [46]. In this study, we used the 3-NP model in brain-isolated mitochondria to investigate, in a selective manner, the role of Complex II on mitochondrial dysfunction and cannabinoid-mediated protection as it has been demonstrated that this model can trigger toxic signals in vitro that involve mitochondrial dysfunction [17].

Hereby, the use of substrates and inhibitors is relevant to stimulate functional responses in mitochondria isolated from cells obtained from the whole brain, as it is known that mitochondrial function is dependent on both the cell environment and oxidation of biomolecules to provide electron carriers to maintain energy metabolism and ETC. In this context, rotenone is useful in preventing the reverse electron transfer and blocking the accumulation of oxaloacetate, thus inhibiting mitochondrial Complex I [43], thereby augmenting ROS formation in neural cells, in contrast to limited production in astrocytes [47]. Consequently, rotenone is a valuable tool to isolate the function of Complex II by preventing the return of electrons to Complex I.

In turn, MTT, and the subsequent formation of formazan, is a technique that has been used as an index of metabolic activity, proliferation, and cell viability in cell cultures and tissue homogenates [48, 49]. In isolated mitochondria, this method has been associated with the mitochondrial reduction capacity in which complexes from the respiratory chain provide their electrons for MTT reduction, and this effect is dependent of dehydrogenases through cofactors such as NADH, FADH, and NAD(P)H, which catalyze the reaction. Therefore, mitochondrial activity has been verified with inhibitors such as rotenone [49, 50]. Our results show that the reduction capacity generated by SDH in the presence of succinate and rotenone was fourfold higher than that of mitochondria in basal conditions, highlighting the relevance of substrates and inhibitors. Notably, when mitochondria were incubated in the absence of rotenone, the mitochondrial reduction capacity was ninefold higher than that of control mitochondria in basal conditions, demonstrating that Complex I plays a crucial role in regulating the reduction capacity in the toxic paradigm evoked by 3-NP, even though this toxin selectively inhibits Complex II.

The partial protective effect evoked by cannabinoids AEA and WIN 55212–2 on the 3-NP-induced Complex II dysfunction was likely to be mediated by activation of mitCB1R, suggesting that mitCB1R completely coordinates the protective function on mitochondrial activity. In this regard, it is known that only 10% of the mitochondrial reduction capacity corresponds to the Complex II activity [50], which in light of our results, reveals the moderate level of contribution of Complex II to this paradigm. Our results also suggest that mitCB1R activation by cannabinoids under conditions of Complex I inhibition only exerts a partial protective effect on the 3-NP decreased reduction capacity; however, when this effect is explored under conditions in which Complex I remains active (without rotenone), the mitCB1R activation-induced protection is complete, suggesting that the activity of Complex I is crucial to orchestrate protective responses, highlighting the possible role of mitCB1R on the regulation of mitochondrial dehydrogenases. Thus, mitCB1R activation under conditions of an active Complex I is likely to generate complete protection against the toxic effect of 3-NP, supporting the concept that this receptor may regulate the functions of Complex I [31, 32]; however, our results suggest that the activity of Complex II might also be regulated by this receptor, either by direct or indirect mechanisms, through the modulation of mitochondrial ion channels [51]. Moreover, it should be considered that results described by other groups demonstrating the regulation of Complex I by mitCB1R have been collected exploring the physiology of this receptor through the assessment of oxygen consumption in mouse mitochondria from hippocampal neurons under basal conditions [31, 32], whereas our study explores the role of this receptor also under pathological conditions in mitochondria isolated from the whole rat brain. This is particularly relevant as it has been demonstrated that mitochondria from neuronal cells and astrocytes exhibit differential functional features [47], where the first are more sensitive to oxidative stress.

It has also been demonstrated that mitCB1R, which is located in the outer membrane of mitochondria, plays a central role in mouse hippocampal neurons treated with the cannabinoid agents WIN 55212–2 and delta9-tetrahydrocannabinol (Δ9-THC) at nanomolar concentrations, producing a reduction in mitochondrial respiration, Complex I activity, protein kinase A (PKA), and cAMP activities, all under physiological conditions. Combined, these effects contribute to the suppression of endocannabinoid-dependent inhibition of depolarization [32]. This regulatory mechanism is crucial for hippocampal physiology through the modulation of neural energy metabolism and the control of cognitive functions; however, in contrast to these effects, our experiments provide a more complete assessment as they explore the effects of cannabinoids and an approach to the possible role of mitCB1R in mitochondria from the whole brain under toxic conditions, using a plethora of endpoints such as assessment of the reduction capacity, ROS formation, and mitochondrial swelling. Accordingly, our study investigates the integrated response of these receptors under pathological conditions, revealing their novel function. Furthermore, a link between mitochondrial activity and the process of memory formation [31] further demonstrated that bioenergetics processes linked to mitCB1R regulate cognitive functions such as memory, which was concluded by demonstrating that these receptors modulate the activity of Complex I through Gαi/o signaling, as well as PKA and adenylate cyclase (AC) inhibition. Although these important findings on the role of the receptor describe major aspects of neural physiology, they impart limited information on the function of neural mitochondria, while our findings provide information on the protective effects evoked by mitochondria obtained from different brain cell types and their responses to a toxic insult.

Although ROS, such as H_2_O_2_, serve as second messengers with fundamental signaling activity in physiological processes such as cell proliferation, mitophagy and apoptosis, synaptic plasticity, and ion channel regulation [5], a deficient regulation in redox homeostasis due to toxic conditions is responsible for the onset and development of neurodegenerative events [52]. Therefore, the relevance of maintaining an optimal mitochondrial energy metabolism lies in the fact that most of ROS formation originated in this organelle. In this work we also evaluated H_2_O_2_ formation induced by 3-NP through the oxidation of DCF. H_2_O_2_ is generated by the reduction of O_2_^·−^ through the activity of manganese superoxide dismutase [42]. In this context, we determined H_2_O_2_ formation produced by selective dysfunction of Complex II in the presence of succinate and antimycin as inhibitors of III_Qi_ site at Complex III to induce reverse transport. Reverse transport directs electrons to Complex I at IQ (the site for ubiquinone reduction), which is the primary origin for O_2_^·−^, reaching IF (site of flavin) where NADH is oxidized to form O_2_^·−^ [53]. The significant increase in H_2_O_2_ induced by 3-NP was decreased by AEA in a likely dependent mitCB1R activation manner, suggesting that regulation of Complex I by this receptor might be responsible for the modulation of ROS formation. We advance this hypothesis based on observations that the toxic formation of ROS was mitigated in this model in a receptor activation-mediated manner, and not by the properties of AEA as a free radical scavenger. Therefore, we hypothesize that this mechanism occurs when, following mitCB1R activation by AEA, inhibition of AC may decrease Complex I activity, inhibiting its subunits [54], among which the site 2 of Fe-S of NADH ubiquinone dehydrogenase (NDFUS2) containing the prosthetic group FeS bound to the reduction site of quinone IQ, which when inhibited, will be unable to provide the electrons needed for O_2_^·−^ production, thus preventing its subsequent reduction to H_2_O_2_, ultimately resulting in a general decrease in ROS formation [55].

Synthetic cannabinoids such as CP55940 and JWH-015 have been shown to exert protective effects against paraquat (Complex I inhibitor) by decreasing ROS formation in rat brain cortical mitochondria due to their properties as free radical scavengers through the presence of hydroxyl groups. These effects were accompanied by attenuation in the membrane potential decrease, also preventing mitochondrial swelling and mitochondrial damage [37]. Despite several cannabinoid compounds, including AEA, have been shown to possess scavenging properties, the fact that mitCB1R activation completely inhibited the ROS formation in this study suggests that mechanisms such as Complex I modulation are more responsible for this protective effect.

Transitory mitochondrial swelling occurs by changes in the volume of mitochondrial matrix and it can be considered a physiological condition for the adequate functioning of mitochondria, as it maintains the ionic concentrations of the cytoplasmic environment, the ETC, and oxidative phosphorylation. The mitochondrial volume is dependent on the fluxes of K^+^ and Ca^2+^ ions through their respective channels, which are located in the inner mitochondrial membrane [56]. The irreversible increase in mitochondrial volume leads to pathological swelling associated with the mPTP opening by loss of inner membrane integrity, triggering changes in mitochondrial morphology in a mechanism linked to the onset of neurodegenerative disorders [17, 56]. However, its role remains poorly understood under physiological conditions, although it has been suggested that it can contribute to the short-term regulation of homeostasis. mPTP opening occurs in response to mitochondrial depolarization induced by Ca^2+^ overload and high levels of ROS formation, and this process uncouples ATP synthase while hydrolyzing ATP, allowing the entrance of ions, water, and solutes with a molecular weight below 1500 Da [57]. 3-NP (5 mM) generates a model of mitochondrial dysfunction by swelling and autophagy, which leads to cell death of SH-SY5Y cells [17]. It has also been tested in rat cortical slices (3-NP 100 µM), where it decreased the mitochondrial reduction capacity while generating cell damage [58]. The decrease in the optical density during the assessment of mitochondrial swelling depends on light transmission, which is proportional to the increase in osmotic pressure following the expansion of the mitochondrial matrix [43, 56]. Given that AEA completely prevented the mitochondrial swelling induced by 3-NP in a likely mitCB1R-dependent manner, we suggest that activation of this receptor might be responsible for modulating mPTP opening through the Gai/o-dependent AC pathway, since this mechanism has been proposed to modulate Ca^2+^ and K^+^ channels [59].

In addition, it has been shown that the synthetic cannabinoids CP55940 and JWH-015 protect cortical rat mitochondria exposed to paraquat [37], where AEA reduced the Ca^2+^-induced cytochrome C release [36]. Our findings support the described evidence, and suggest the involvement of a cannabinoid signaling in the prevention of mitochondrial swelling, though our results suggest that this signal may be mediated by activation of mitCB1R and this process can be linked to the prevention of ROS formation and the subsequent preservation of the mitochondrial reduction capacity.

Our study also suggests that CB1R signaling is analogous in the cytoplasmic and mitochondrial membranes (inhibition of AC and possible regulation of Ca^2+^ and K^+^ channels) [59]. The activation of mitCB1R might regulate the activity of Ca2 + transport, favoring the decrease of Ca^2+^ influx and preventing the Ca^2+^-dependent mPTP opening, thus providing a novel potential mechanism of mitochondrial protection.

Of major relevance is the fact that in vivo evidence supports the concept that CB1R activation by cannabinoids affords protection to the CNS against the alterations induced by 3-NP. The intrastriatal lesion with this toxin in rats decreased the expression of mRNA for CB1R [60]. In CB1R KO mice (the N171-82Q strain), the striatal lesion with 3-NP worsened motor performance and pathological damage of brain tissue [61]. In addition, the use of the selective CB1R agonists arvanil improved motor activity in 3-NP–lesioned rats [62]. In turn, WIN 55212–2 (i.p.) prevented motor alterations, oxidative stress, neural damage, and loss of CB1R expression in rats intrastriatally lesioned with 3-NP [46]. Moreover, the i.p. administration of a well-known inhibitor of fatty acid amide hydrolase (FAAH, the degrading enzyme for AEA), URB597, to rats prevented motor alterations and cell damage in the same toxic model [63]. As a whole, this evidence provides solid support to our present in vitro findings at the mitochondrial level, and generates an important physiological and physiological correlate between the results of this study and those collected from in vivo studies.

Finally, in this study, we used 500 nM of AEA and WIN 55212–2 for most of our experiments, and these concentrations were obtained from our own response-effect curves. While it is known that AEA Ki for CB1R is 61 nM and WIN 55212–2 Ki for CB1R is 62.3 nM [64], we rather used the optimal concentrations of these compounds found in our experimental conditions as little is known yet about the specific properties of mitCB1R. It should be considered that no other forms of cannabinoid receptors have been reported in mitochondria, while these agents have been commonly used to investigate CB1R responses [31, 32]. Our data are also supported by the effect exerted by the selective CB1R antagonist AM281, tested herein at a concentration near its Ki (12 nM) [65], with optimal concentration also obtained from our own concentration curve (Fig. 3A).

Combined, the findings of this study provide new insight into the role of the ECS in the regulation of brain mitochondrial activity under physiological and pathological conditions. Hence, future studies should address whether targeted manipulation of this pathway may afford therapeutic strategy for neurodegenerative diseases.

## Concluding Remarks

In summary, we report that the use of cannabinoids and the possible mitCB1R activation exert protective effects against the toxic actions induced by 3-NP in rat brain mitochondria. These properties include the decrease in ROS formation, the reduction of mitochondrial swelling and the subsequent preservation of mitochondrial reduction capacity. Our results support the hypothesis that mitCB1R regulates mitochondrial energy metabolism, ETC, and activity of dehydrogenases, thus modulating ROS formation derived from the inhibition of mitochondrial Complex II. In the toxic paradigm used herein, the ECS’s dual and adaptive character at the mitochondrial level was evidenced. The findings of the present work provide novel evidence for the consideration of cannabinoid-based therapies for the treatment of neurodegenerative disorders with mitochondrial dysfunction components.

**Fig. 7:**
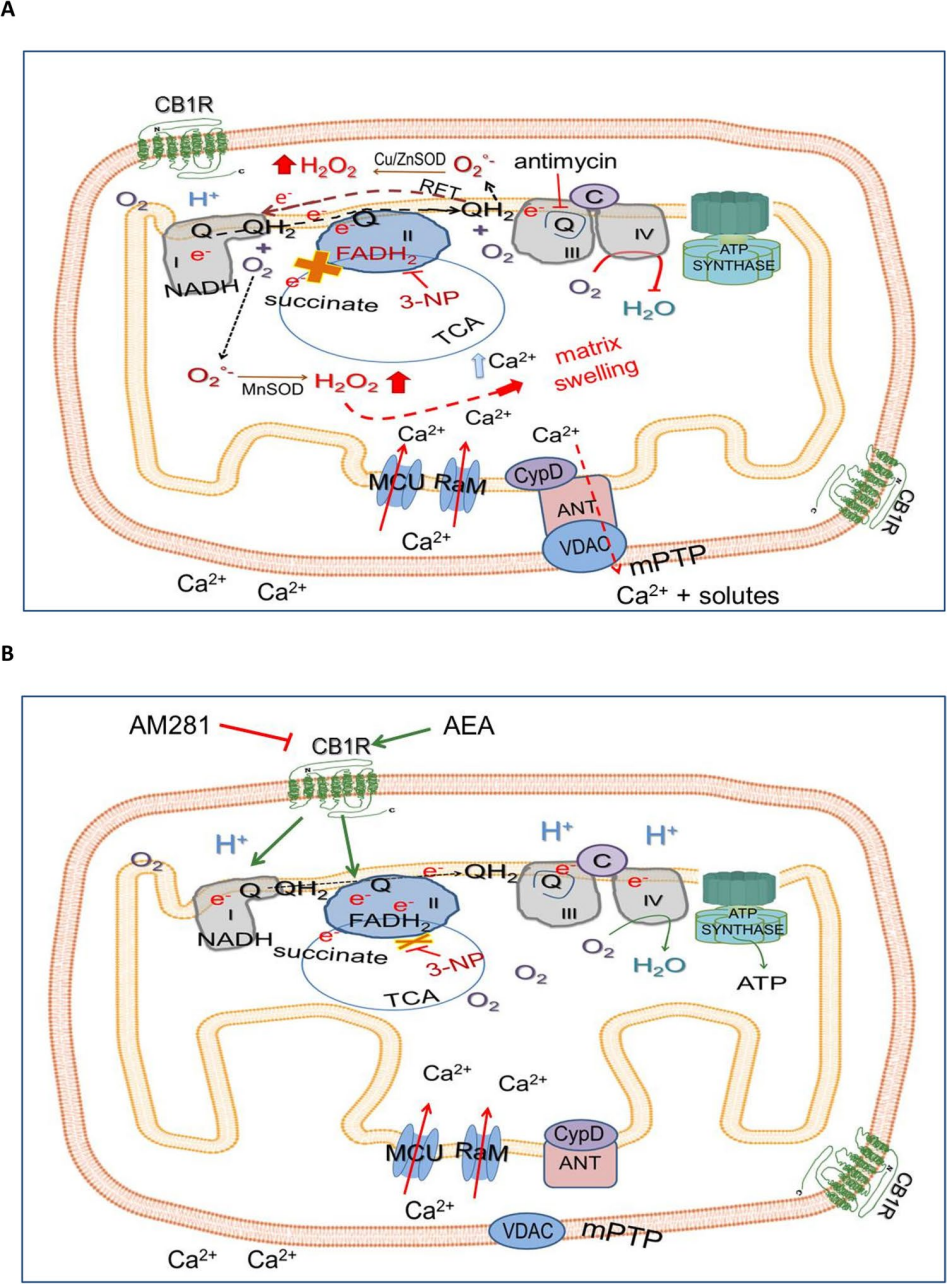
Schematic representation of the effects of the neurotoxin 3-nitropropionic acid (3-NP), the endocannabinoid anandamide (AEA), and the selective antagonist of cannabinoid receptor 1 AM281, in mitochondria isolated from the rat brain

Figure 7 summarizes the series of events occurring in the experimental paradigm investigated in this study. Figure 7A summarizes the events linked to mitochondrial damage induced by 3-NP [16, and partially demonstrated hereby]. This toxin competes with succinate for the enzyme succinate dehydrogenase (SDH, Complex II) due to the structural similarity between these two compounds. 3-NP covalently binds SDH, thereby blocking its function, reducing the mitochondrial respiratory chain activity and increasing the hydrogen peroxide (H_2_O_2_) production. This species is formed by reduction of superoxide anion (O_2_°^−^) through the activity of the enzyme superoxide dismutase, which is present both in its manganese-dependent (Mn-SOD) form in the mitochondrial matrix, and in its copper/zinc-dependent (Cu/Zn-SOD) form in the intermembrane space. The sites known for the production of O_2_°^−^ in mitochondria are the flavin site and the ubiquinone site in Complex I, the flavin site in Complex II, and the binding site for quinol in Complex III. When complexes remain reduced, electron leakage augments, thus increasing the formation of reactive oxygen species (ROS). The addition of antimycin (a well-known mitochondrial Complex III inhibitor) to mitochondria increases the levels of H_2_O_2_ in the presence of 3-NP through the reverse electron transport (RET) on the Q pool [53]. Increased ROS levels, together with a mitochondrial Ca^2+^ overload in the matrix through the rapid mode of calcium uptake (Ram) and the mitochondrial calcium uniporter (MCU) channels, induce the mitochondrial permeability transition pore (mPTP) opening, thus allowing the entrance of water and solutes, and provoking mitochondrial swelling. In turn, the mPTP is composed of cyclophilin D (CyD, located in mitochondrial matrix), the adenine nucleotide translocase (ANT, located in the inner mitochondrial membrane), and the voltage-dependent Ca^2+^-channel (VDAC, located in the outer mitochondrial membrane) [56]. Figure 7B depicts the protective effect of AEA on the 3-NP–induced toxic events in mitochondria probably through the activation of mitCB1R (demonstrated hereby). Pretreatment of isolated mitochondria with AEA induced the activation of mitCB1R, which attenuated the alterations produced by 3-NP on the mitochondrial respiratory chain activity, inhibiting H_2_O_2_ formation and mitochondrial swelling induced by Ca^+2^ overload. This scheme proposes that mitCB1R activation prevents the 3-NP-induced toxicity by regulating the function of the respiratory chain, specifically of Complex I and Complex II. The involvement of mitCB1R in this model was evidenced by the blockade of these receptors by the selective antagonist AM281.

### Supplementary Information

Below is the link to the electronic supplementary material.

Supplementary file1 (DOCX 203 KB)

## Data Availability

The datasets used and/or analyzed during the current study are available from the corresponding authors upon reasonable request.

## Abbreviations

MTT: 1-(4,5-Dimethylthiazol-2-yl)-3,5-diphenylformazan
3-NP: 3-Nitropropionic acid
AEA: Anandamide
ANOVA: Analysis of variance
cAMP: Cyclic AMP
CB1R: Cannabinoid Receptor 1
CB2R: Cannabinoid Receptor 2
DCF: Dihydrodichlorofluorescein
ECS: Endocannabinoid system
ETC: Electron transport chain
GPR55: G55 protein coupled receptor
H_2_O_2_: Hydrogen peroxide
HD: Huntington’s disease
mitCB1R: Mitochondrial CB1 receptor
mPTP: Mitochondrial permeability transition pore
O_2_^·^^−^: Superoxide radical
PKA: Protein Kinase A
ROS: Reactive oxygen species
SDH: Succinate dehydrogenase
TRPV1: Vanilloid Transient Receptor Potential V1
