# Correction: Anandamide and WIN 55212–2 Afford Protection in Rat Brain Mitochondria in a Toxic Model Induced by 3-Nitropropionic Acid: an In Vitro Study

**DOI:** 10.1007/s12035-024-04125-4

**Published:** 2024-03-25

**Authors:** Marisol Maya-López, Luis Angel Monsalvo-Maraver, Ana Laura Delgado-Arzate, Carolina I. Olivera-Pérez, Mohammed El-Hafidi, Alejandro Silva-Palacios, Omar Medina-Campos, José Pedraza-Chaverri, Michael Aschner, Alexey A. Tinkov, Isaac Túnez, Socorro Retana-Márquez, Cecilia Zazueta, Abel Santamaría

**Affiliations:** 1 Doctorado en Ciencias Biológicas y de La Salud, Universidad Autónoma Metropolitana, 09310 Mexico City, Mexico; 2 Facultad de Ciencias, Universidad Nacional Autónoma de México, 04510 Mexico City, Mexico; 3 Departamento de Biomedicina Cardiovascular, Instituto Nacional de Cardiología, SSA, 14080 Mexico City, Mexico; 4 Laboratorio F-315, Departamento de Biología, Facultad de Química, Universidad Autónoma de México, 04510 Mexico City, Mexico; 5 Department of Molecular Pharmacology, Albert Einstein College of Medicine, Bronx, NY 10461 USA; 6 Laboratory of Molecular Dietetics, IM Sechenov First Moscow State Medical University (Sechenov University), Moscow, 119435 Russia; 7 Department of Human Ecology and Bioelementology, and Department of Medical Elementology, Peoples’ Friendship University of Russia (RUDN University), Moscow, 117198 Russia; 8 Instituto de Investigaciones Biomedicas Maimónides de Córdoba (IMIBIC), Córdoba, Spain; 9 Departamento de Bioquímica y Biología Molecular, Facultad de Medicina y Enfermería, Universidad de Córdoba, Córdoba, Spain; 10 Red Española de Excelencia en Estimulación Cerebral (REDESTIM), 14071 Córdoba, Spain; 11 Departamento de Biología de La Reproducción, Universidad Autónoma Metropolitana-Iztapalapa, 09310 Mexico City, Mexico

**Correction: Molecular Neurobiology**

10.1007/s12035-024-03967-2

The original version of this article unfortunately contained error in affiliation 1.

The error lies in the Department name of affiliation number 1 of the first author to where "Programa de Posgrado" should be "Doctorado" to read "Doctorado en Ciencias Biológicas y de La Salud, Universidad Autónoma Metropolitana, 09310 Mexico City, Mexico".

The original article has been corrected.

