# Supplementary material for: Anandamide and WIN 55212–2 Afford Protection in Rat Brain Mitochondria in a Toxic Model Induced by 3-Nitropropionic Acid: an In Vitro Study
Source: Mol Neurobiol. 2024 Feb 3;61(9):6435–52. doi: 10.1007/s12035-024-03967-2 (PMC11338978; doi:10.1007/s12035-024-03967-2)
Supplement: Supplementary file 1 — Supplementary file1 (DOCX 203 KB) [file 12035_2024_3967_MOESM1_ESM.docx]

| **State 3** | **State 4** | **Respiratory Control**  **Index** |
| --- | --- | --- |
| Complex I | | |
| 45.2 | 21.6 | 2.1 |
| Complex II | | |
| 43.2 | 14.04 | 6.4 |

**Supplementary Figure 1:** Respiratory control index (ngAtO/min/mg protein) determined to demonstrate mitochondrial coupling, using the Clark type electrode (Yellow Springs Instruments, Yellow Springs, OH, USA). Substrates for Complex I (5 mM malate plus 3 mM glutamate) and Complex II (10 mM succinate plus 1 μM rotenone as a Complex I inhibitor) were used to activate oxygen consumption in a medium containing 125 mM KCl, 10 mM HEPES and 3 mM inorganic phosphorus (Pi) at pH 7.3. Recordings started by adding 2 mg mitochondrial protein to determine the baseline oxygen consumption (state 4), and mitochondrial respiration was evaluated 1 min later by adding 300 nmol ADP to determine the respiration associated to ATP production (state 3). The respiratory control rate (state 3/state 4) was assessed to demonstrate that mitochondrial respiration is coupled to oxidative phosphorylation with a value greater than 1.


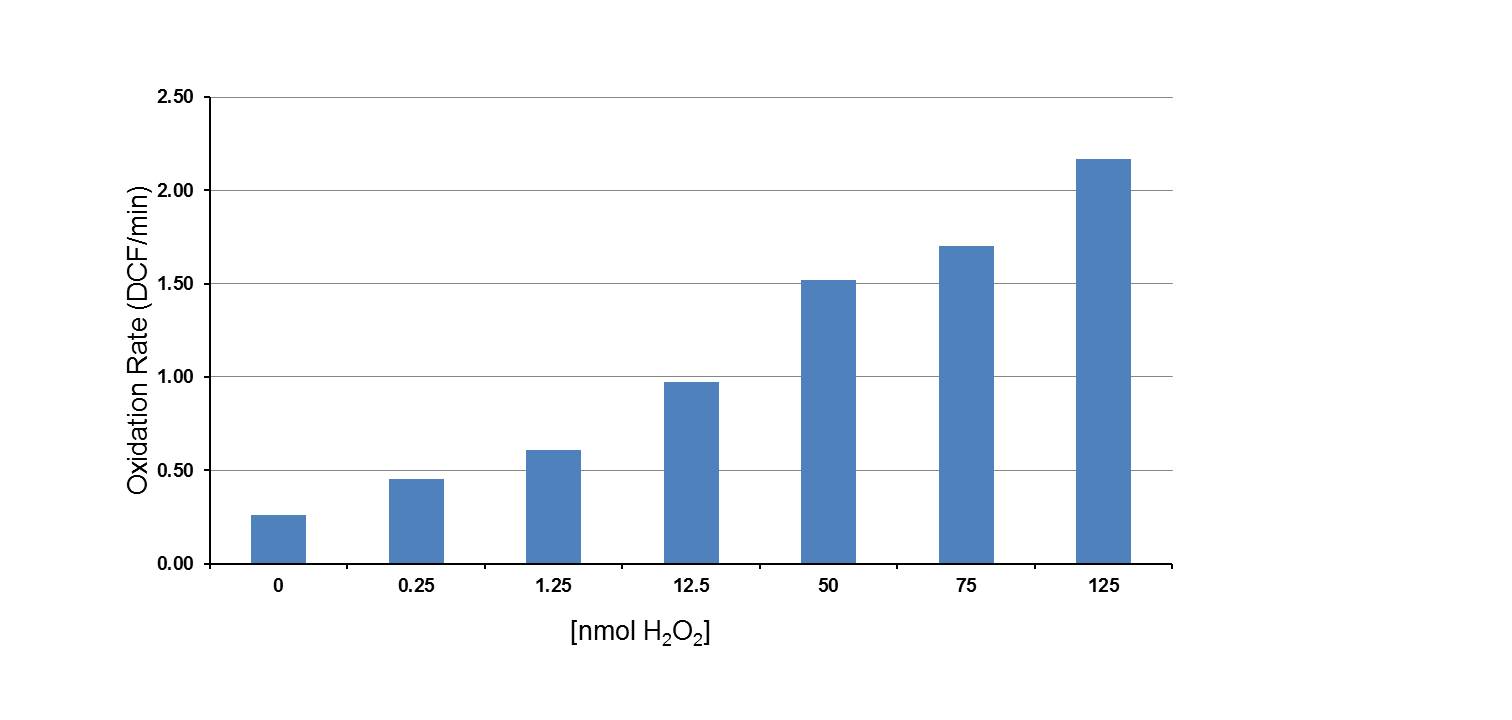


**Supplementary Figure 2:** H_2_O_2_ standard curve (nmol/oxidation rate of DCF-H/min). A concentration standard curve of H_2_O_2_ [0 to 500 μM] was carried out using 100 µg mitochondrial protein. Mitochondria were prepared with horseradish peroxidase (PR: 1 μg) plus protonated dichlorofluorescein (DCF-H: 1 µM). Assessment of fluorescence was recorded in time, where after the first min, H_2_O_2_ was added and measurements were taken for 15 min. The standard curve (μM) was transformed into nmol to quantify H_2_O_2_ production in pmol H_2_O_2_/min/mg protein.


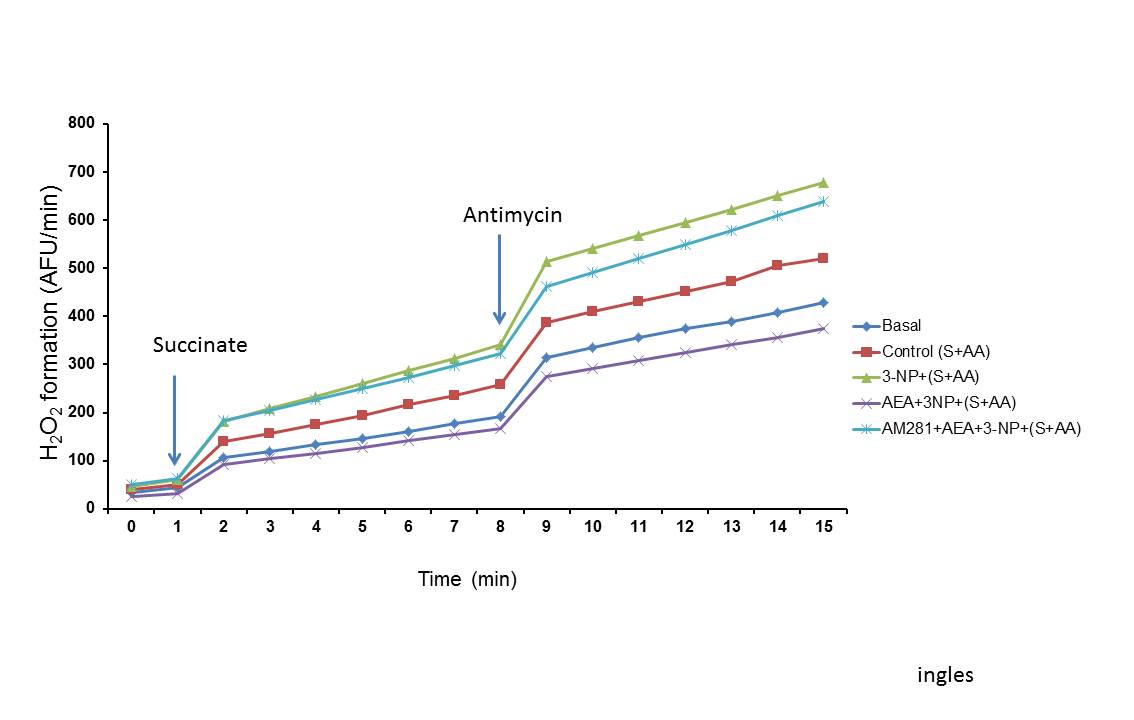


**Supplementary Figure 3.** Graph represents H_2_O_2_ formation assessed as fluorescence of protonated dichlorofluorescein as arbitrary fluorescence units (AFU) with respect to time. Mitochondria were added with horseradish peroxidase (HP) and protonated dichlorofluorescein (DCF-H). Once initiated the fluorescence recording, succinate was added during the first min to stimulate H_2_O_2_ production and detection, and 7 min later, antimycin, a well-known Complex III inhibitor, was added to enhance H_2_O_2_ formation.

**4A**


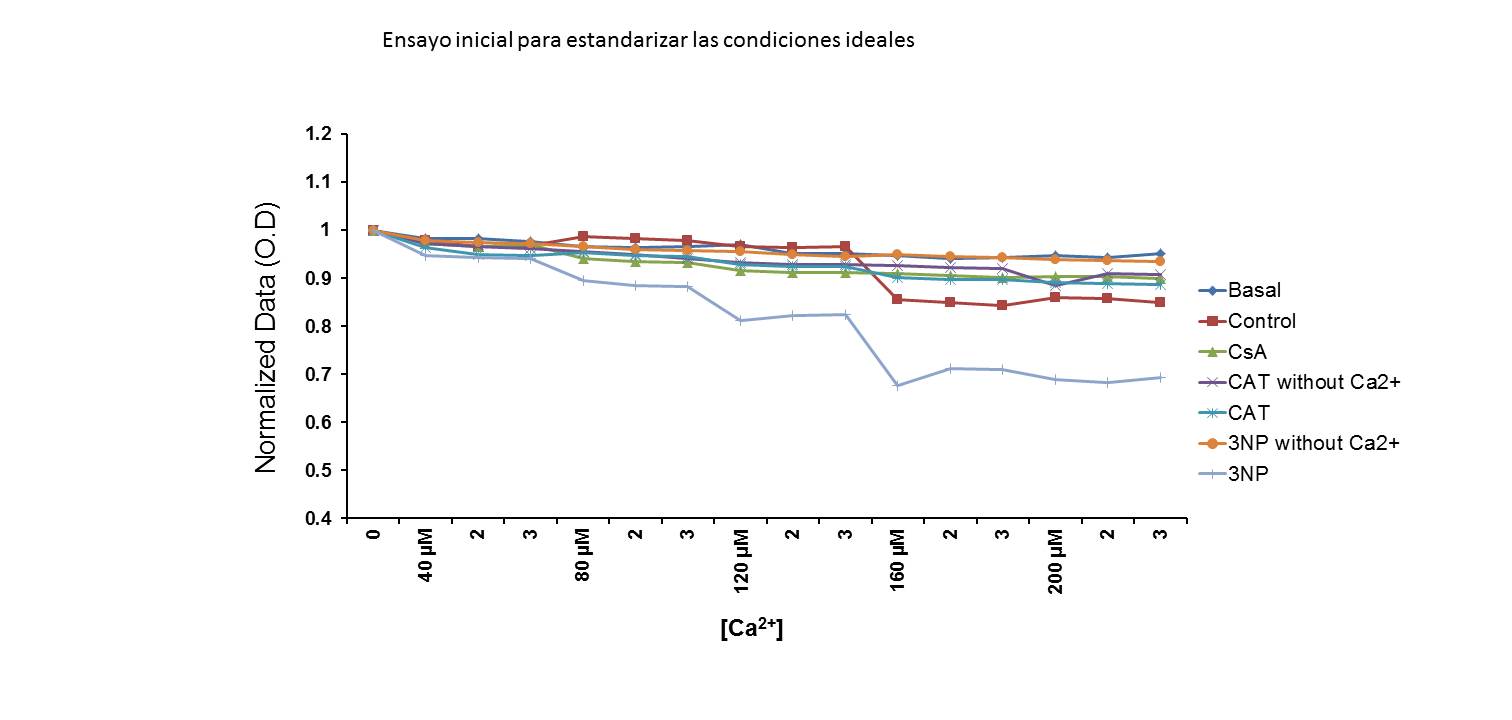


**4B**


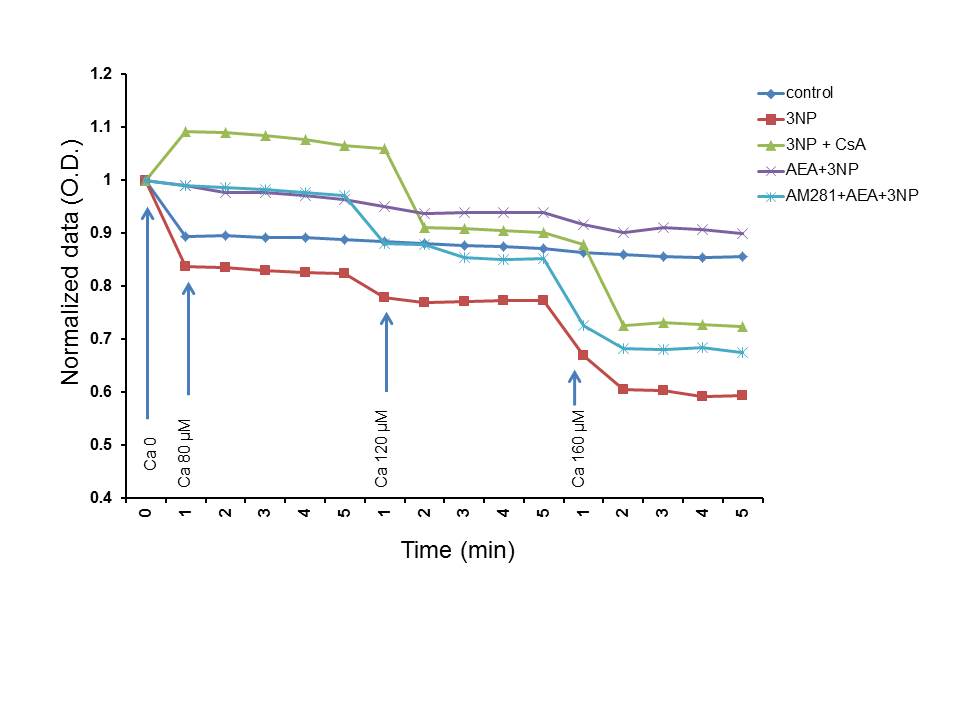


**Supplementary Figures 4A and 4B.** Both plot graphs represent mitochondrial swelling induced by increased Ca^2+^ concentrations. In **A**, 3-Nitropropionic acid (3-NP) acts as a mitochondrial swelling inducer in the presence of increased Ca^2+^ concentrations as plot line decreases constantly, whereas 1 μM carboxytractylosyde (CAT), a well-known ADP/ATP translocase (ANT or adenine nucleotide translocator protein, a protein forming part of the structure of mPTP) inhibitor did not induce mitochondrial swelling. In **B**, the preventive effect of AEA on 3-NP-induced mitochondrial swelling is depicted, where the response induced by AEA is mediated by mitRCB1, evidenced by the effect of the antagonist AM281. Cyclosporin A closed mPTP when interacted with cyclophilin D, which is located in the mitochondrial matrix**.**
